# Detecting susceptibility genes in case-control studies using set association

**DOI:** 10.1186/1471-2156-4-S1-S9

**Published:** 2003-12-31

**Authors:** Sung Kim, Kui Zhang, Fengzhu Sun

**Affiliations:** 1 Molecular and Computational Biology Program, Department of Biological Sciences, University of Southern California, Los Angeles, California, USA; 2 Department of Biostatistics, University of Alabama at Birmingham, Birmingham, Alabama, USA

## Abstract

Complex diseases are generally caused by intricate interactions of multiple genes and environmental factors. Most available linkage and association methods are developed to identify individual susceptibility genes assuming a simple disease model blind to any possible gene - gene and gene - environmental interactions. We used a set association method that uses single-nucleotide polymorphism markers to locate genetic variation responsible for complex diseases in which multiple genes are involved. Here we extended the set association method from bi-allelic to multiallelic markers. In addition, we studied the type I error rates and power for both approaches using simulations based on the coalescent process. Both bi-allelic set association (BSA) and multiallelic set association (MSA) tests have the correct type I error rates. In addition, BSA and MSA can have more power than individual marker analysis when multiple genes are involved in a complex disease. We applied the MSA approach to the simulated data sets from Genetic Analysis Workshop 13. High cholesterol level was used as the definitive phenotype for a disease. MSA failed to detect markers with significant linkage disequilibrium with genes responsible for cholesterol level. This is due to the wide spacing between the markers and the lack of association between the marker loci and the simulated phenotype.

## Background

Current beliefs hold that complex diseases are generally caused by multiple genetic variation. However, most available linkage and association methods are based on the assumption that a single genetic variation is primarily responsible for the disease under study. Only a few approaches considered interactions of multiple genes and environmental factors in identifying susceptibility loci for complex diseases [1-3].

Hoh et al. [1] developed a set association approach to identify genetic variation responsible for complex diseases when multiple genes are involved. They applied bi-allelic set association (BSA) to a real data set and were able to identify several single-nucleotide polymorphisms (SNPs) of interest that were in linkage disequilibrium (LD) with susceptibility locus for restenosis, the re-blockage of the coronary artery after treatment. However, they did not study the type I error rate and power of their approach. Another issue is that the set association method of Hoh et al. [1] can only be applied to bi-allelic markers.

The organization of the paper is as follows. We first briefly describe BSA and provide an intuitive framework for the extension from BSA to multiallelic set association (MSA) as well as a description of the various simulated data sets. Next, we study the type I error and power of the BSA and MSA using simulated data sets based on coalescence with an underlying disease model. Finally, we present the results from applying MSA to Genetic Analysis Workshop 13 simulated data sets without knowledge of the answers to the longitudinal data generated by Daw et al. [4].

## Methods

### BSA

For completeness, we briefly describe the BSA method of Hoh et al. [1] for use in genome-wide association studies based on bi-allelic markers, such as SNPs. The proposed statistic is based on allelic association (AA) and Hardy-Weinberg Disequilibrium (HWD). Consider a case-control study design with *n *cases and *n *controls genotyped at a marker locus with *k *alleles. A 2 × *k *contingency table is constructed and a statistic of AA is then computed as follows:



where *p*_*s *_and *q*_*s *_are the frequency of the allele *s *for the cases and controls, respectively. The above statistic has an approximate χ^2 ^distribution with *k*-1 degrees of freedom.

HWD [5], which also has an approximate χ^2 ^distribution with *k*(*k *- 1)/2 degrees of freedom, is then calculated among the cases according to the following statistic:



where *q*_*s *_and *q*_*t *_are the frequencies of alleles *s *and *t*, respectively, while *p*_*ss *_and *p*_*st *_are the frequencies of genotypes *ss *and *st*, respectively. For BSA, *k *= 2.

Hoh et al. [1] suggested that extremely high HWD values at a locus might indicate genotyping errors at the locus. Hence, they suggested trimming the *d *largest case-based HWD values to 0, thereby removing these problematic loci. *d *is empirically determined from the number of loci whose control-based HWD statistic is greater than , the β quantile of the standard χ^2 ^distribution with one degree of freedom. However it should also be noted that both AA and HWD values are high if the locus is in strong LD with one of the disease variants.

Now suppose that there are *m *bi-allelic marker loci. Hoh et al. [1] . defined a new statistic *s*_*i *_= *AA*_i _* *HWD*_*i*_, where *AA*_*i *_and *HWD*_*i *_are the AA and HWD statistic for marker locus *i*, 1 ≤ *i *≤ *m*. The values *s*_1_, *s*_2_,...,*s*_*m *_are then ordered from the largest to the smallest with *s*_(1) _≥ *s*_(2) _≥ ... ≥ *s*_(*m*)_. Then they defined



Next, Monte Carlo permutations were used to find the *p*-value of BSA. The collection of cases and controls are permuted 1000 times and for each permutation, *j*, an analogous sum statistic  is calculated. Let *p*_*i *_be the fraction of times that  is smaller than *S*_*i*_. The minimum of *p*_*i*_,



is treated as the final statistic.

To evaluate the overall significance, the above process is repeated such that for the *j*^th ^permuted case-control data set,



is obtained. The overall significance level, *P*_*overall*_, can be approximated by the fraction of times that *P*_*j *_is smaller than *P*. The null hypothesis of no association of the region with the disease is rejected if *P*_*overall *_is less than a pre-specified type I error, for example, 0.05.

### MSA

BSA cannot be directly applied to multiallelic markers because the χ^2 ^statistics might have varying degrees of freedom for markers with different number of alleles. Therefore, BSA is extended to consider multiallelic markers. For each marker locus, *i*, calculate the χ^2 ^statistics, *AA*_*i*_, based on the allele frequencies in the cases and the controls, and *HWD*_*i *_from cases. The corresponding *p*-values are then obtained for AA, *p*_*AA*_(*i*), and HWD, *p*_*HWD*_(*i*), on the basis of χ^2 ^approximation, respectively.

Trimming was conducted similar to the method of Hoh et al. [1]. However, the *d *smallest HWD *p*-values were removed from MSA analysis along with the corresponding *p*_*AA*_. The remaining *p*-values for AA and HWD were then multiplied and the products were used as a score denoted as *s*_*i*_, that is,

*s*_*i *_= *p*_*AA *_(*i*) * *p*_*HWD *_(*i*).

The scores for all the markers are sorted from the smallest to the largest such that *s*_(1) _≤ *s*_(2) _≤ ... ≤ *s*_(*m*)_. We define a sum statistic similar to BSA as



Finally, the *p*-value was again determined by Monte Carlo permutations.

### Bi-allelic coalescent simulations

The coalescent theory, first introduced by Kingman [6], allowed the inference of genealogies from the observed genotypic data. The process was to take extant *k *individuals and trace backward in time to a common ancestor [6]. Unlike Kingman, who considered no crossover events, Hudson [7] and Kaplan and Hudson [8] provided the framework to consider the coalescence with a constant population recombination rate θ. Once the genealogy is determined, SNPs are added using an infinite-many-sites model with population mutation rate μ. This model assumes that mutation rates occur uniformly along the region of interest and that each mutation generates a novel SNP that does not already exist in the population.

We simulated 2000 haplotypes consisting of a large number of consecutive SNPs across a genomic region using the above coalescent process with recombination. θ and μ were both set to 200, which correspond to a 200-kb genomic region of human DNA on the basis of a large survey of the human genome. This strategy has been used by Nordborg and Tavaré [9].

One hundred cases and 100 controls with genotypes for approximately 130 adjacent polymorphic sites were sampled from this simulated population of haplotypes according to the following disease model. A disorder with two disease genes was considered. We assumed that the first disease gene to be located between the 35^th ^and the 45^th ^marker loci and the second disease gene to be located between the 75^th ^and the 85^th ^marker loci. The frequency of the high-risk allele of the first disease gene and the second disease gene is denoted as ε and φ, respectively. ε and φ were set to be either 0.1 ± 0.05 or 0.2 ± 0.05 throughout the simulation. The first condition constrains the location of the disease locus to be isolated to two specified regions while the second condition constrains the disease allele frequency. If no such marker loci exist, the sample set is discarded [10].

Consider the phenotypic aspect of the disease model with *D*_*i*_, *i *= 1, 2 as the high-risk allele and *d*_*i*_, *i *= 1, 2 as the low risk allele, respectively. The haplotype risks for *d*_1_*d*_2_,*D*_1_*d*_2_, *D*_2_*d*_1_, and *D*_1_*D*_2 _were assumed to be δ, δλ, δλ, and δλ^2^, respectively, where δ is the phenocopy rate and λ is the genotype relative risk. Furthermore, a haplotype multiplicative disease model was assumed, i.e., the penetrance of an individual equals the product of the two corresponding haplotype risks. As an example, if the two haplotypes of an individual are *D*_1_*D*_2_/*D*_1_*d*_2_, the penetrance for the individual is δλ^2 ^* δλ = δ^2^λ^3 ^[10].

We drew 100 times from the simulated population of haplotypes with replacement to generate a sample set, each containing 100 cases and 100 controls. The entire process of generating a population of haplotypes and sample sets was repeated 10 additional times giving a total of 1000 simulated data sets. By modifying the above parameters, the data sets created under various conditions allowed the exploration of type I error and power for individual marker analysis, BSA, and MSA.

### Multiallelic simulations

To conduct the multiallelic simulations, we utilized the haplotypes generated in the previous section. Here, we considered overlapping two-locus haplotypes as alleles for a single marker locus. Thus, we have a set of multiallelic marker loci with at most four alleles at each marker locus. Finally, 100 cases and 100 controls were also generated according to the same procedure and disease model as previously described.

### GAW 13 data set

Based on the Framingham Heart Study, the simulated data sets attempted to model the observed longitudinal family data with 399 genome-wide microsatellite markers and 50 trait genes. In addition to genetic effects, environmental covariates (smoking and alcohol consumption), along with hypertension diagnosis and treatment were used to simulate the observed phenotypes. The pedigree structure was generated to be nearly identical to the Framingham Heart Study with varying degrees of heritability of eight longitudinal quantitative traits [4].

The first measured cholesterol level was chosen as the phenotype of interest. The empirical distribution of cholesterol level in a replicate set was approximated from the cholesterol levels of all the individuals. Next, the upper 15% and lower 15% quantiles were then estimated from this observed distribution. We considered an extreme sampling design in which an individual was considered affected if the first measured cholesterol level exceeded the upper limit and unaffected if it was below the lower limit. Cases and controls were randomly selected based on these measured cholesterol levels with no two members from the same family. We required that there were no missing genotypes for the selected individuals.

## Results

### Type I error rates and power of BSA

Table 1 illustrates the results of the test with individual marker analysis versus BSA for different values of ε, φ, δ, and λ based on 1000 replicate sets. We first studied the type I error rates with λ = 1. When λ = 1 (the third and fourth columns), all the haplotypes were assumed to have the same penetrance, creating a population where any detectable association indicates a false positive result.

The values of type I error rates for individual marker method based on AA were calculated by the number of replicates containing at least one locus with a significant result divided by the total number of replicate sets. Bonferroni correction was used to adjust for multiple comparisons. Type I error rates for BSA with trimming at  was also determined by the number of replicates with significant results divided by total number of replicate sets. The statistical significance level α was set to 0.05. As shown, the estimated type I error rates were close to the true pre-specified type I error of 5%.

For the power study of BSA, the parameters δ and λ were set to create two complex disease scenarios. The power of the tests should increase as the penetrance λ increases. The power of both individual marker analysis and BSA increases. When comparing the sixth column with the fifth column (also the eighth column with the seventh column) in Table 1, BSA is much more powerful than individual marker analysis when multiple genes are involved in a disease.

### Type I error rates and power of MSA

As shown in Table 2, individual marker analysis using AA versus MSA based on 1,000 multiallelic replicate sets had the same conclusions compared with the results using BSA for bi-allelic replicate sets (Table 1). Evaluation of type I error rates with λ = 1 (third and fourth column) were close to the pre-specified type I error of 5%. MSA outperformed AA for both test cases when λ = 2 and λ = 4.

### Applications to the simulated data of GAW 13

All of the 11 different replicates of the simulated data from GAW13 used for MSA resulted in the failure to reject the null hypothesis of no association as shown in Table 3. Replicate 26 is presented as a typical outcome from the application of MSA.

The upper 15% quantile was calculated at 210 mg/dl and the lower 15% quantile was estimated to be 155 mg/dl. For replicate 26, we obtained 125 cases and 105 controls. Based on individual marker analysis using AA, 14 out of the 399 genome-wide markers had a *p*-value less than 0.05. However, it should be noted that even if no markers were associated with cholesterol level, an average of 20 markers should show significant results using type I error of 0.05. The 14 markers most likely represented false positive errors and a Bonferroni correction would correct this inflation. The MSA was applied to the data using AA × HWD with trimming threshold set at . An overall non-significant *p*-value *P*_*overall *_= 0.5417 was obtained. For the other 10 replicates, MSA showed no significant association with *P*_*overall *_ranging from 0.08 to 0.94.

## Discussion

In this paper, we developed a new approach for genome-wide association studies with multi-allelic markers based on BSA. There are several advantages for MSA over any individual marker analysis. First, it is more powerful than individual marker analysis, which is consistent with conclusions of BSA in Hoh et al. [1]. Second, the approach can theoretically be applied with any reasonable score function at each locus, which can allow us to combine different kinds of data, such as SNPs joined with microsatellite markers, to gain higher power in analysis. With the accumulation of data, genome-wide association studies can benefit from this method.

MSA was applied to 11 simulated GAW13 data sets and no significant associations were found. The result is expected given that the average marker spacing is approximately 8.5 cM and that there were no associations (between the quantitative trait and marker loci) modeled into the simulation. Following typical disease mapping techniques, once a linkage study is conducted to locate potential susceptibility sites, MSA could then be used to detect associations after more markers are genotyped.

## Figures and Tables

**Table 1:** The approximate type I error rates and power of individual marker analysis using AA versus BSA based on 1000 simulated data sets for different values of ε, φ, δ, and λ

		**δ = 1, λ = 1**		**δ = 0.20, λ = 2**		**δ = 0.05, λ = 4**	
		
ε	φ	**Individual Marker**	**BSA**	**Individual Marker**	**BSA**	**Individual Marker**	**BSA**
0.1	0.1	0.06	0.03	0.083	0.326	0.557	0.860
0.1	0.2	0.06	0.04	0.230	0.610	0.686	0.956

**Table 2:** The approximate type I error rates and power of individual marker analysis using AA versus MSA based on 1000 simulated data sets for different values of ε, φ, δ, and λ

		**δ = 1, λ = 1**		**δ = 0.20, λ = 2**		**δ = 0.05, λ = 4**	
		
ε	φ	**Individual Marker**	**MSA**	**Individual Marker**	**MSA**	**Individual Marker**	**MSA**
0.1	0.1	0.04	0.055	0.138	0.333	0.863	0.936
0.1	0.2	0.04	0.055	0.411	0.622	0.973	0.991

**Table 3:** Results of MSA analysis for 11 replicate sets from GAW13

**Replicate**	**Number of Cases**	**Number of Controls**	**85% quantile (mg/ml)**	**15% quantile (mg/ml)**	***P*_*overall*_**
1	132	121	210	156	0.443
2	142	92	211	156	0.945
3	124	103	210	157	0.802
4	141	112	211	157	0.082
5	150	103	210	157	0.112
6	140	105	209	155	0.148
7	125	128	210	155	0.481
8	146	111	209	156	0.095
9	124	121	210	154	0.694
10	142	94	209	156	0.744
26	125	105	210	155	0.542
